# Severe acute pancreatitis and toxic hepatitis after solitary dietary supplement ingestion: A case report

**DOI:** 10.1097/MD.0000000000050060

**Published:** 2026-08-07

**Authors:** Yeonwoo Kim, Woo Young Nho

**Affiliations:** a Department of Emergency Medicine, CHA Gumi Medical Center, CHA University, Gumi, Republic of Korea; b Department of Emergency Medicine, Keimyung University Dongsan Hospital, Daegu, Republic of Korea.

**Keywords:** acute pancreatitis, catechin, drug-induced pancreatitis, green tea

## Abstract

**Rationale:**

Although many products have been approved for weight loss, their efficacy remains debatable owing to the insufficient scientific evidence in the relevant literature. Furthermore, because of the limited clinical data and research, the adverse effects of these products remain unelucidated.

**Patient concerns:**

A 36-year-old woman presented to the emergency department with generalized weakness. She had a personal history of undergoing weight reduction trials several times. For 2 weeks, she ingested only water and dietary supplements containing green tea catechin and no other food or drugs.

**Diagnoses:**

Laboratory examination results indicated highly elevated liver and pancreatic enzyme levels. Radiological examinations indicated severe acute pancreatitis and the possibility of severe fatty liver or toxic hepatitis. Furthermore, she was clinically diagnosed with substance-induced acute pancreatitis and was suspected to have concomitant toxic hepatitis.

**Interventions:**

Intensive medical therapy was started, including intravenous hydration, prophylactic antibiotics, and nutritional balance, along with psychiatric support. With adequate management, both clinical manifestations and laboratory results improved.

**Outcomes:**

Finally, she was discharged 15 days after admission without any obvious complications.

**Lessons:**

Most of the adverse events reported to be associated with catechin are related to the gastrointestinal tract and particularly lead to hepatic injury. Interestingly, catechin is known for its protective effects on the pancreas. This report highlights the possibility of catechin-induced pancreatitis, in contrast to the generally accepted protective effects on the pancreas.

## 1. Introduction

The desire to lose weight due to body dissatisfaction has been recognized as a long-standing problem.^[1]^ Unfortunately, this desire has been further aggravated owing to the weight stigma associated with obesity.^[2]^ Furthermore, unrealistic standards of beauty-driven media have affected women in all age groups.^[1]^ According to a previous study, one-third of adults use dietary supplements for weight reduction.^[3]^ Notably, recent global trends indicate a surge in unregulated and falsified products within the dietary supplement market.^[4]^ With the increasing consumption of dietary supplements among young women, normal-weight people have also hopped on this trend.^[5]^ Although more than 300 types of weight loss products have been approved to date, their effectiveness remains controversial because of insufficient scientific evidence in the literature.^[3]^ In addition, owing to the limited clinical data and research on the subject, their side effects have not been elucidated. Plant- and herb-derived compounds constitute a substantial proportion of dietary supplements. Bioactive substances from fruits, nuts, or tea leaves have demonstrated health benefits, including antioxidant effects.^[6–8]^However, these substances may occasionally pose risks to the gastrointestinal tract or liver.^[8,9]^ Green tea leaves are a well-known dietary supplement ingredient valued for their health benefits, but concerns regarding their adverse effects have focused primarily on hepatotoxicity.^[10–15]^ Herein, we report a case of severe acute pancreatitis following a 2-week ingestion of a solitary dietary supplement containing green tea catechin.

## 2. Case report

**Figure 1.**
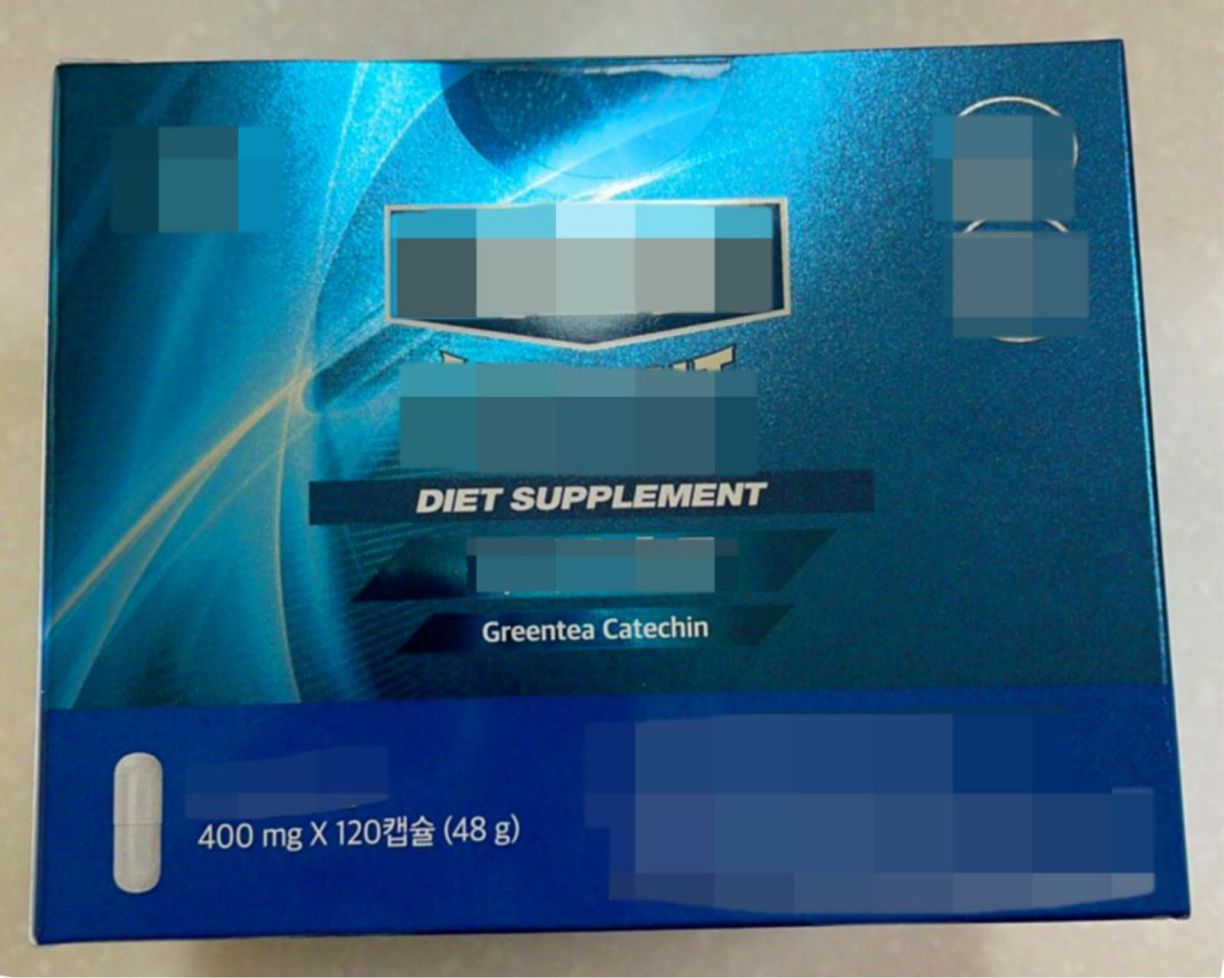
Image of the dietary health supplement. CE, CT = computed tomography, FFS, FOV, GT, Im, Idx, kVp, mAs, Se, TI, TB.

**Figure 2.**
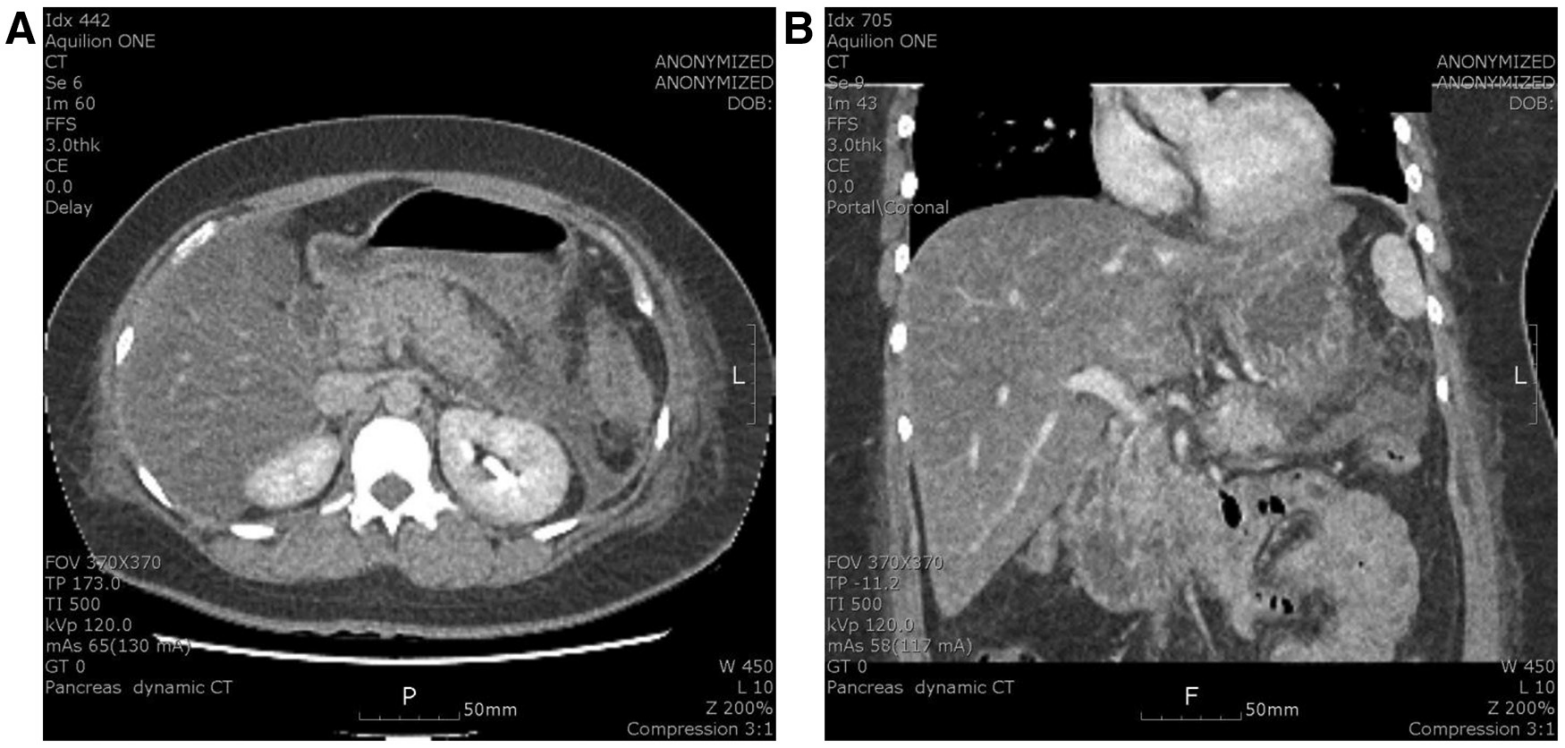
Computed tomography scan. (A) A diffuse parenchymal swelling with peripancreatic infiltration can be seen. Fluid collection with subtle peripheral enhancement is observed around the tail portion of the pancreas, peri-splenic space, and left anterior pararenal space. Furthermore, a heterogeneously decreased parenchymal enhancement can be seen in the distal body and tail portion of the pancreas. (B) Hypo-attenuated liver indicates the possibility of severe fatty liver or toxic hepatitis. CE, CT = computed tomography, FFS, FOV, GT, Im, Idx, kVp, mAs, Se, TI, TB.

**Figure 3.**
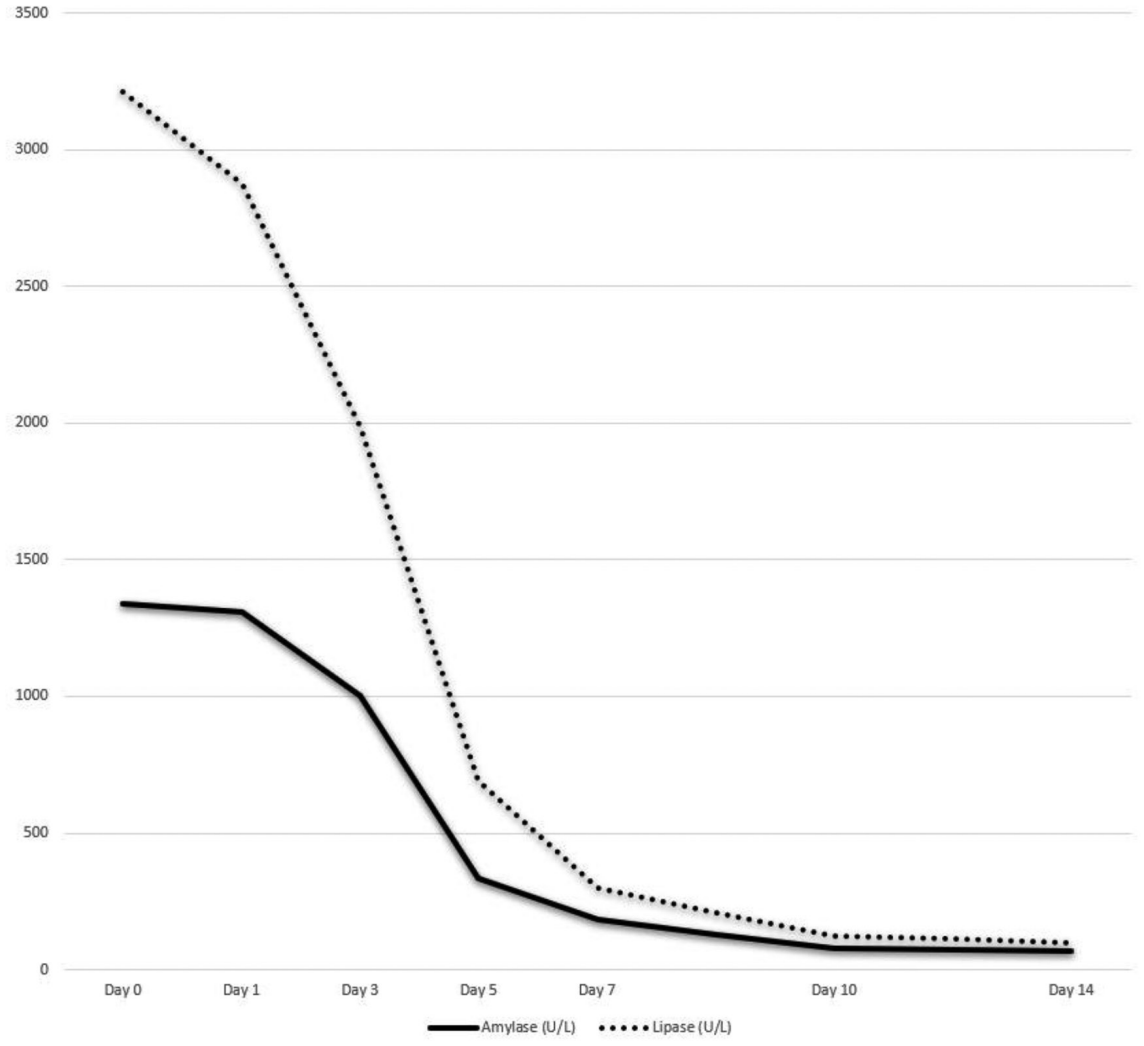
Trend of pancreatic enzyme levels during the course of treatment.

A 36-year-old woman presented to the emergency department (ED) with generalized weakness and polydipsia persisting for several days. Her medical history indicated major depression and intermittent explosive disorder. The patient reported that because her body mass index was 27 kg/m^2^, she had attempted to lose weight several times previously. Two weeks ago, she again tried losing weight using a dietary supplement (commercial name: Max Cut) containing 300 mg of green tea catechin per daily dose (Fig. 1). Notably, she did not consume any food or drugs during this period, except for water and the abovementioned dietary supplement. Approximately 1 week before her visit to the ED, she started experiencing generalized weakness. Moreover, for the last 3 days, she drank more than 5 L of water. On arrival at the ED, the patient exhibited lethargy and slurred speech, and her vital signs were as follows: blood pressure, 90/60 mm Hg; pulse rate, 76 beats/min; and respiratory rate, 12 breaths/min. Her physical examination revealed a moderately distended abdomen with tenderness in the right upper quadrant. Neurological examination revealed no definite focal neurological defect. Her laboratory results indicated highly elevated liver and pancreatic enzyme levels. Moreover, her aspartate aminotransferase, alanine aminotransferase, alkaline phosphatase, gamma-glutamyl transferase, serum amylase, and serum lipase levels were 150, 185, 155, 113, 1338, and 3124 IU/L, respectively. Electrolyte imbalance was indicated by serum sodium and potassium levels of 118 and 4.5 mEq/L, respectively. In contrast, other results, including viral markers and lipid profiles, were unremarkable. Computed tomography (CT) scans revealed diffuse parenchymal swelling with peripancreatic infiltration. Moreover, fluid collection with subtle peripheral enhancement was observed around the tail portion of the pancreas, perisplenic space, and left anterior pararenal space. Furthermore, heterogeneously decreased parenchymal enhancement was noted in the distal body and tail portion of the pancreas (Fig. 2A). No cholelithiasis or bile duct stones were detected. Based on the CT findings, the radiologist suggested the presence of severe acute pancreatitis, which was compatible with CT grade D and a CT severity index of 7. In addition, the finding of hypoattenuated liver indicated the possibility of severe fatty liver or toxic hepatitis (Fig. 2B). The Roussel Uclaf Causality Assessment Method score for drug-induced liver injury was calculated as 8, indicating a probable causal relationship. Finally, the patient was diagnosed with substance-induced acute pancreatitis along with suspected concomitant toxic hepatitis. Intensive medical therapy was subsequently initiated, including intravenous hydration, prophylactic intravenous carbapenem (500 mg every 8 hours for 7 days), and parenteral nutritional support for the first 10 days, along with psychiatric support. Psychiatric consultation excluded intentional overdose and attributed the presentation to substance misuse for weight reduction. With adequate management, both clinical manifestations and laboratory results improved (Fig. 3). Finally, she was discharged 15 days after admission without any obvious complications, and a 3-month outpatient follow-up visit revealed no delayed sequelae.

## 3. Discussion

Drug-induced pancreatitis is an exceptional condition that is difficult to diagnose. Moreover, its classification involves reviewing evidence from human case reports, ruling out other causes, and finding the results of rechallenge.^[16,17]^ Furthermore, the category of substance-induced pancreatitis remains unestablished owing to the difficulty in investigating the connection between the disease and the abovementioned factors. This report presents a novel finding of catechin-induced pancreatitis. In addition, it contributes as a milestone in diagnosing substance-induced pancreatitis based on obvious clinical circumstances and the exclusion of other possible causes.

Extensive research has been conducted on catechin since it was first isolated in the early 20th century.^[18]^ A previous study reported that catechin is found in abundance in green tea, cocoa, and some fruits; in particular, green tea catechins majorly comprise epigallocatechin-3-gallate (EGCG).^[19]^ Notably, EGCG is known as an antioxidant and is clinically used in cancer therapy. Furthermore, it is known to exert anti-infective effects, even against coronavirus disease 2019.^[19–21]^ Another clinical property of EGCG is its ability to target lipid metabolism. Although its exact mechanism has not been established to date, some studies have proven that it inhibits lipid absorption in the gastrointestinal tract, thereby decreasing adipose tissue accumulation and fat oxidation during rest or exercise.^[22,23]^ Owing to its benefits for fat reduction, the EGCG compound has been commonly used in dietary products for weight loss.^[3,5]^ Thus, EGCG has become one of the main ingredients for the so-called fat burner supplements.^[23]^ Given the presence of controversy between its risks and benefits, adverse effects associated with the ingestion of green tea extracts, including EGCG, have largely been reported.^[13,14]^ Notably, hepatotoxicity has been frequently recognized in both human and animal studies.^[13–15]^ In addition, a positive relationship has been observed between the EGCG dose and its toxicological effects, which were more severe in the fasting state.^[13,14]^ Additional toxicological effects, such as mutagenicity, genotoxicity, reproductive and developmental toxicity, and carcinogenicity, were identified in an animal study.^[13]^ Gastrointestinal irritation and hepatotoxicity were the most commonly reported effects in human studies, and other side effects may follow. To the best of our knowledge, no clinical study has proven pancreatic toxicity due to EGCG in humans.^[13]^

Interestingly, EGCG is known for its protective effects on the pancreas. Moreover, previous studies have demonstrated its preventive effects against pancreatic fibrosis and cancer, its counteracting effect on stress-induced pancreatitis, and its preservative role in pancreatitis.^[24–27]^ Unfortunately, in the present case, we could not determine the exact mechanism of this paradoxical situation. Prolonged fasting may cause liver damage, and we hypothesize that dehydration or metabolic shifts may have amplified EGCG toxicity. Possible mechanisms include EGCG’s pro-oxidant effects at high doses or fasting-induced dysregulation of pancreatic autophagy. Some observational studies have reported that fasting increases the prevalence of acute pancreatitis; however, the evidence on this remains insufficient, even though fasting has long been considered a good therapy for acute pancreatitis.^[28]^

This report has several limitations despite the strength of the causality inferred from exclusive ingestion. First, a rechallenge test was not performed for ethical reasons; consequently, the Badalov/Wolfe classification was estimated as class IV. We suspect potential underreporting of similar cases in Asia, where green tea consumption is high, and further evidence and clinical experience are needed to substantiate this pattern of toxicity. Second, the plasma EGCG level was not measured, as the in-hospital laboratory did not support EGCG testing, and the patient declined external testing owing to the high cost. Third, although other possible causes of hepatic injury were excluded, liver biopsy was not performed to confirm the diagnosis.

## 4. Conclusion

We report a case of severe acute pancreatitis following a 2-week ingestion of a solitary dietary supplement containing green tea catechin. This report highlights the possibility of catechin-induced pancreatitis in contrast to the generally accepted protective effects on the pancreas. Policy implications include advocating for EGCG labeling warnings, such as avoiding intake while fasting, as well as increased clinician education regarding this potential adverse effect.

## 5. Patient perspective

By the time she got to the emergency room, she was terrified. She had been feeling incredibly low about her body image and desperate for a quick change, which led her to purchase a weight-loss supplement online. Because it was marketed as a “natural supplement,” she genuinely believed it was safe. Her doctor told her this was a condition that usually only happens to chronic alcoholics or older people. When the medical team traced the timeline back to the weight-loss supplement, she felt a wave of intense guilt and shame – she had inadvertently poisoned her own body. She now understands how completely unregulated and dangerous the supplement industry can be, especially for young people looking for a quick fix.

## Author contributions

**Writing – original draft:** Yeonwoo Kim.

**Conceptualization:** Woo Young Nho.

**Supervision:** Woo Young Nho.

**Writing – review & editing:** Yeonwoo Kim, Woo Young Nho.
